# Bis[4-(dimethyl­amino)pyridinium] penta­bromidochloridostannate(IV)

**DOI:** 10.1107/S160053680901705X

**Published:** 2009-05-14

**Authors:** Yong Jang, Kong Mun Lo, Seik Weng Ng

**Affiliations:** a Department of Chemistry, University of Malaya, 50603 Kuala Lumpur, Malaysia

## Abstract

In the title compound, (C_7_H_11_N_2_)_2_[SnBr_5_Cl], there is Br/Cl disorder in 0.6561 (12):0.3439 (12) and 0.8438 (12):0.1561 (12) ratios over two of three halide sites in the centrosymmetric anion, such that an overall formulation of [SnBr_5_Cl]^2−^ arises. In the crystal, associations of two cations and one anion linked by N—H⋯Br hydrogen bonds occur.

## Related literature

For related 4-dimethyl­amino­pyridinium halogenoorgano­stannates, see: Lo & Ng (2008 ▶); Norhafiza *et al.* (2008 ▶); Yau *et al.* (2008 ▶).
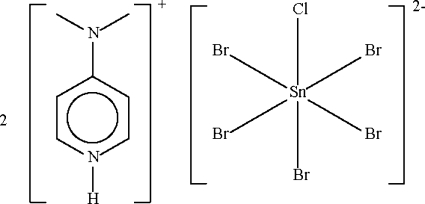


## Experimental

### 

#### Crystal data

(C_7_H_11_N_2_)_2_[SnBr_5_Cl]
*M* *_r_* = 800.05
Monoclinic, 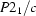

*a* = 8.4424 (1) Å
*b* = 11.8821 (2) Å
*c* = 11.8868 (2) Å
β = 107.123 (1)°
*V* = 1139.55 (3) Å^3^
*Z* = 2
Mo *K*α radiation
μ = 10.01 mm^−1^
*T* = 100 K
0.30 × 0.10 × 0.10 mm

#### Data collection

Bruker SMART APEX CCD diffractometer
Absorption correction: multi-scan (*SADABS*; Sheldrick, 1996 ▶) *T*_min_ = 0.152, *T*_max_ = 0.434 (expected range = 0.129–0.367)
9261 measured reflections
2613 independent reflections
2408 reflections with *I* > 2σ(*I*)
*R*_int_ = 0.022

#### Refinement

*R*[*F* ^2^ > 2σ(*F* ^2^)] = 0.021
*wR*(*F* ^2^) = 0.053
*S* = 1.01
2613 reflections
121 parameters
4 restraints
H-atom parameters constrained
Δρ_max_ = 0.91 e Å^−3^
Δρ_min_ = −0.95 e Å^−3^

### 

Data collection: *APEX2* software (Bruker, 2007 ▶); cell refinement: *SAINT* (Bruker, 2007 ▶); data reduction: *SAINT*; program(s) used to solve structure: *SHELXS97* (Sheldrick, 2008 ▶); program(s) used to refine structure: *SHELXL97* (Sheldrick, 2008 ▶); molecular graphics: *X-SEED* (Barbour, 2001 ▶); software used to prepare material for publication: *publCIF* (Westrip, 2009 ▶).

## Supplementary Material

Crystal structure: contains datablocks global, I. DOI: 10.1107/S160053680901705X/hb2965sup1.cif

Structure factors: contains datablocks I. DOI: 10.1107/S160053680901705X/hb2965Isup2.hkl

Additional supplementary materials:  crystallographic information; 3D view; checkCIF report

## Figures and Tables

**Table 1:** Selected bond lengths (Å). *X* = (Br, Cl)

Sn1—*X*1	2.5608 (3)
Sn1—*X*2	2.5618 (3)
Sn1—*X*3	2.5687 (3)

**Table 2:** Hydrogen-bond geometry (Å, °)

*D*—H⋯*A*	*D*—H	H⋯*A*	*D*⋯*A*	*D*—H⋯*A*
N1—H1⋯Br1	0.88	2.47	3.327 (2)	165

**Fig. 1.**
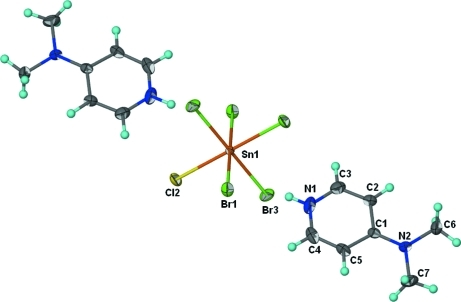
The molecular structure of (I) viewed at the 70% probability level. Hydrogen atoms are drawn as spheres of arbitrary radius.

## supplementary crystallographic information

### Experimental

Dibenzyltin dichloride (0.37 g, 1 mmol) and 4-dimethylaminopyridine hydrobromide perbromide (0.73 g, 2 mmol) were heated in chloroform for 3 hours. Colourless blocks of (I) separated from the cool solution after a day. The crystal structure showed that the benzyl groups on tin had been cleaved in the reaction.

### Refinement

Hydrogen atoms were placed at calculated positions (C–H 0.95, N–H 0.88 Å) and were treated as riding on their parent atoms, with *U*(H) set to 1.2 times *U*_eq_(C,*N*).

Two of the three halogen atoms in the stannate are disordered. The pair of Br1/Cl1 and Br2/Cl2 atoms initially refined to nearly 1.5Br and 0.5Cl atoms; the total occupancy of the disordered bromine atoms was then fixed as exactly 1.5. The occupancy of the disordered chlorine atoms was similarly set to be exactly 0.5.

The U^ij^ values of the Br1 and Cl1 atoms were restrained to be identical, as were those of the Br2 and Cl2 atoms.

### Crystal data

**Table:**

(C_7_H_11_N_2_)_2_[SnBr_5_Cl]	*F*(000) = 752
*M**_r_* = 800.05	*D*_x_ = 2.332 Mg m^−^^3^
Monoclinic, *P*2_1_/*c*	Mo *K*α radiation, λ = 0.71073 Å
Hall symbol: -P 2ybc	Cell parameters from 5777 reflections
*a* = 8.4424 (1) Å	θ = 2.5–28.3°
*b* = 11.8821 (2) Å	µ = 10.01 mm^−^^1^
*c* = 11.8868 (2) Å	*T* = 100 K
β = 107.123 (1)°	Block, colourless
*V* = 1139.55 (3) Å^3^	0.30 × 0.10 × 0.10 mm
*Z* = 2	

### Data collection

**Table:**

Bruker SMART APEX CCD diffractometer	2613 independent reflections
Radiation source: fine-focus sealed tube	2408 reflections with *I* > 2σ(*I*)
graphite	*R*_int_ = 0.022
ω scans	θ_max_ = 27.5°, θ_min_ = 2.5°
Absorption correction: multi-scan (*SADABS*; Sheldrick, 1996)	*h* = −10→10
*T*_min_ = 0.152, *T*_max_ = 0.434	*k* = −15→15
9261 measured reflections	*l* = −15→15

### Refinement

**Table:**

Refinement on *F*^2^	Primary atom site location: structure-invariant direct methods
Least-squares matrix: full	Secondary atom site location: difference Fourier map
*R*[*F*^2^ > 2σ(*F*^2^)] = 0.021	Hydrogen site location: inferred from neighbouring sites
*wR*(*F*^2^) = 0.053	H-atom parameters constrained
*S* = 1.01	*w* = 1/[σ^2^(*F*_o_^2^) + (0.0276*P*)^2^ + 1.7721*P*] where *P* = (*F*_o_^2^ + 2*F*_c_^2^)/3
2613 reflections	(Δ/σ)_max_ = 0.001
121 parameters	Δρ_max_ = 0.91 e Å^−^^3^
4 restraints	Δρ_min_ = −0.95 e Å^−^^3^

### Fractional atomic coordinates and isotropic or equivalent isotropic displacement parameters (Å^2^)

**Table:**

	*x*	*y*	*z*	*U*_iso_*/*U*_eq_	Occ. (<1)
Sn1	0.5000	0.5000	0.5000	0.01035 (7)	
Br1	0.50979 (5)	0.63602 (3)	0.66902 (3)	0.02104 (10)	0.6561 (12)
Br2	0.58402 (4)	0.33881 (2)	0.64884 (3)	0.02209 (9)	0.8438 (12)
Br3	0.80725 (3)	0.53820 (3)	0.52248 (3)	0.02568 (8)	
Cl1	0.50979 (5)	0.63602 (3)	0.66902 (3)	0.02104 (10)	0.3439 (12)
Cl2	0.58402 (4)	0.33881 (2)	0.64884 (3)	0.02209 (9)	0.1561 (12)
N1	0.6520 (3)	0.8746 (2)	0.5886 (3)	0.0271 (6)	
H1	0.5999	0.8123	0.5962	0.032*	
N2	0.9139 (3)	1.15682 (19)	0.5545 (2)	0.0186 (5)	
C1	0.8253 (3)	1.0669 (2)	0.5659 (2)	0.0154 (5)	
C2	0.7373 (4)	1.0025 (2)	0.4666 (3)	0.0194 (5)	
H2	0.7358	1.0257	0.3898	0.023*	
C3	0.6551 (4)	0.9077 (2)	0.4809 (3)	0.0245 (6)	
H3	0.5991	0.8642	0.4139	0.029*	
C4	0.7277 (4)	0.9358 (3)	0.6848 (3)	0.0251 (6)	
H4	0.7203	0.9125	0.7595	0.030*	
C5	0.8137 (4)	1.0296 (2)	0.6768 (3)	0.0211 (6)	
H5	0.8670	1.0711	0.7459	0.025*	
C6	0.9127 (4)	1.1990 (3)	0.4393 (3)	0.0249 (6)	
H6A	0.9488	1.1393	0.3955	0.037*	
H6B	0.9883	1.2633	0.4490	0.037*	
H6C	0.8002	1.2229	0.3958	0.037*	
C7	1.0105 (4)	1.2208 (3)	0.6571 (3)	0.0291 (7)	
H7A	0.9353	1.2650	0.6887	0.044*	
H7B	1.0866	1.2715	0.6336	0.044*	
H7C	1.0740	1.1687	0.7175	0.044*	

### Atomic displacement parameters (Å^2^)

**Table:**

	*U*^11^	*U*^22^	*U*^33^	*U*^12^	*U*^13^	*U*^23^
Sn1	0.00978 (11)	0.01195 (11)	0.00936 (12)	−0.00163 (8)	0.00287 (9)	−0.00006 (8)
Br1	0.0306 (2)	0.01735 (16)	0.01761 (19)	−0.00301 (13)	0.01094 (15)	−0.00399 (12)
Br2	0.03025 (18)	0.01759 (15)	0.01582 (17)	−0.00058 (12)	0.00271 (13)	0.00430 (11)
Br3	0.01302 (14)	0.03789 (17)	0.02652 (17)	−0.00656 (11)	0.00643 (12)	−0.00107 (12)
Cl1	0.0306 (2)	0.01735 (16)	0.01761 (19)	−0.00301 (13)	0.01094 (15)	−0.00399 (12)
Cl2	0.03025 (18)	0.01759 (15)	0.01582 (17)	−0.00058 (12)	0.00271 (13)	0.00430 (11)
N1	0.0210 (12)	0.0210 (12)	0.0407 (17)	−0.0003 (10)	0.0115 (11)	0.0092 (11)
N2	0.0175 (11)	0.0195 (11)	0.0167 (12)	−0.0033 (9)	0.0019 (9)	−0.0018 (9)
C1	0.0123 (12)	0.0183 (12)	0.0145 (13)	0.0038 (9)	0.0025 (10)	0.0005 (10)
C2	0.0183 (13)	0.0212 (13)	0.0170 (14)	0.0007 (10)	0.0024 (11)	−0.0009 (11)
C3	0.0208 (14)	0.0230 (14)	0.0265 (16)	−0.0016 (11)	0.0019 (12)	−0.0023 (12)
C4	0.0214 (14)	0.0320 (15)	0.0256 (16)	0.0108 (12)	0.0130 (12)	0.0122 (13)
C5	0.0207 (14)	0.0283 (14)	0.0141 (14)	0.0061 (11)	0.0049 (11)	0.0018 (11)
C6	0.0240 (14)	0.0240 (14)	0.0251 (16)	−0.0053 (11)	0.0047 (12)	0.0069 (12)
C7	0.0290 (16)	0.0304 (16)	0.0233 (17)	−0.0097 (12)	0.0007 (13)	−0.0110 (13)

### Geometric parameters (Å, °)

**Table:**

Sn1—Br1	2.5608 (3)	C1—C2	1.419 (4)
Sn1—Cl1^i^	2.5608 (3)	C1—C5	1.421 (4)
Sn1—Br1^i^	2.5608 (3)	C2—C3	1.360 (4)
Sn1—Br2	2.5618 (3)	C2—H2	0.9500
Sn1—Cl2^i^	2.5618 (3)	C3—H3	0.9500
Sn1—Br2^i^	2.5618 (3)	C4—C5	1.348 (4)
Sn1—Br3^i^	2.5687 (3)	C4—H4	0.9500
Sn1—Br3	2.5687 (3)	C5—H5	0.9500
N1—C4	1.347 (4)	C6—H6A	0.9800
N1—C3	1.347 (4)	C6—H6B	0.9800
N1—H1	0.8800	C6—H6C	0.9800
N2—C1	1.334 (3)	C7—H7A	0.9800
N2—C6	1.456 (4)	C7—H7B	0.9800
N2—C7	1.464 (4)	C7—H7C	0.9800
			
Br1—Sn1—Cl1^i^	180.0	C1—N2—C6	121.5 (2)
Br1—Sn1—Br1^i^	180.0	C1—N2—C7	121.6 (2)
Cl1^i^—Sn1—Br1^i^	0.000 (11)	C6—N2—C7	116.9 (2)
Br1—Sn1—Br2	89.520 (11)	N2—C1—C2	121.3 (3)
Cl1^i^—Sn1—Br2	90.480 (11)	N2—C1—C5	122.5 (3)
Br1^i^—Sn1—Br2	90.480 (11)	C2—C1—C5	116.2 (3)
Br1—Sn1—Cl2^i^	90.480 (11)	C3—C2—C1	120.3 (3)
Cl1^i^—Sn1—Cl2^i^	89.520 (11)	C3—C2—H2	119.8
Br1^i^—Sn1—Cl2^i^	89.520 (11)	C1—C2—H2	119.8
Br2—Sn1—Cl2^i^	180.000 (12)	N1—C3—C2	120.9 (3)
Br1—Sn1—Br2^i^	90.480 (11)	N1—C3—H3	119.6
Cl1^i^—Sn1—Br2^i^	89.520 (11)	C2—C3—H3	119.6
Br1^i^—Sn1—Br2^i^	89.520 (11)	N1—C4—C5	121.1 (3)
Br2—Sn1—Br2^i^	180.000 (12)	N1—C4—H4	119.4
Cl2^i^—Sn1—Br2^i^	0.00 (2)	C5—C4—H4	119.4
Br1—Sn1—Br3^i^	89.512 (11)	C4—C5—C1	120.6 (3)
Cl1^i^—Sn1—Br3^i^	90.488 (11)	C4—C5—H5	119.7
Br1^i^—Sn1—Br3^i^	90.488 (11)	C1—C5—H5	119.7
Br2—Sn1—Br3^i^	90.316 (10)	N2—C6—H6A	109.5
Cl2^i^—Sn1—Br3^i^	89.684 (10)	N2—C6—H6B	109.5
Br2^i^—Sn1—Br3^i^	89.684 (10)	H6A—C6—H6B	109.5
Br1—Sn1—Br3	90.488 (11)	N2—C6—H6C	109.5
Cl1^i^—Sn1—Br3	89.512 (11)	H6A—C6—H6C	109.5
Br1^i^—Sn1—Br3	89.512 (11)	H6B—C6—H6C	109.5
Br2—Sn1—Br3	89.684 (10)	N2—C7—H7A	109.5
Cl2^i^—Sn1—Br3	90.316 (10)	N2—C7—H7B	109.5
Br2^i^—Sn1—Br3	90.316 (10)	H7A—C7—H7B	109.5
Br3^i^—Sn1—Br3	180.0	N2—C7—H7C	109.5
C4—N1—C3	120.8 (3)	H7A—C7—H7C	109.5
C4—N1—H1	119.6	H7B—C7—H7C	109.5
C3—N1—H1	119.6		
			
C6—N2—C1—C2	5.6 (4)	C4—N1—C3—C2	1.3 (4)
C7—N2—C1—C2	−177.4 (3)	C1—C2—C3—N1	1.7 (4)
C6—N2—C1—C5	−174.8 (3)	C3—N1—C4—C5	−2.4 (4)
C7—N2—C1—C5	2.2 (4)	N1—C4—C5—C1	0.6 (4)
N2—C1—C2—C3	176.4 (3)	N2—C1—C5—C4	−177.5 (3)
C5—C1—C2—C3	−3.3 (4)	C2—C1—C5—C4	2.2 (4)

Symmetry codes: (i) −*x*+1, −*y*+1, −*z*+1.

### Hydrogen-bond geometry (Å, °)

**Table:**

*D*—H···*A*	*D*—H	H···*A*	*D*···*A*	*D*—H···*A*
N1—H1···Br1	0.88	2.47	3.327 (2)	165
